# Maternal Age and Inadequate Prenatal Care in West Virginia: A Project WATCH Study

**DOI:** 10.13023/jah.0601.03

**Published:** 2024-09-01

**Authors:** Madelin Gardner, Amna Umer, Brian Hendricks, Toni Marie Rudisill, Candice Lefeber, Collin John, Christa Lilly

**Affiliations:** West Virginia University; West Virginia University; West Virginia University; West Virginia University

**Keywords:** Appalachia, maternal age, prenatal care

## Abstract

**Introduction:**

Adequate prenatal care (PNC) is essential to the overall health of mother and infant. Teen age and advanced maternal age (AMA) are known risk factors for poor birth outcomes. However, less is known about whether these age groups are associated with inadequate PNC.

**Purpose:**

This study sought to determine the potential association between maternal age (in groups, aged 20–24, 25–29, 30–34, 35–39, and >40) and inadequate PNC (visits).

**Methods:**

West Virginia (WV) Project WATCH population-level data (May 2018–March 2022) were used for this study. Multiple logistic regressions were performed on inadequate PNC (less than 10 visits) with maternal age categories, adjusting for covariates including maternal race, smoking status, substance use status, parity, education, geographic location, and insurance status.

**Results:**

Results demonstrate that both young and AMA pregnant people are more likely to receive inadequate PNC. PNC is particularly important for these groups, as they are at increased risk of poor birth outcomes. Just over 11% of pregnant people who gave birth in WV received inadequate PNC. Participants aged 19 years and younger (aOR:1.3, CI:(1.2,1.4)), 35–39 years (aOR:1.1, CI:(1.0,1.2)), and 40 years (aOR:1.3, CI:(1.1,1.5)) were at increased odds of inadequate PNC relative to 25–29-year-olds.

**Implications:**

Results indicate that easily obtained demographics, such as a pregnant person’s age, can be utilized by policymakers and clinical interventionists to improve birth outcomes by increasing PNC outreach for these groups.

## INTRODUCTION

The National Center for Health Statistics states that in the period 2016–2020, approximately 15% of women in the U.S. annually received inadequate prenatal care (PNC) based on the Adequacy of Prenatal Care Utilization (APNCU) index.^1^,^2^ Receiving adequate PNC is essential to the health of mother and infant.^2^–^8^ Typical PNC involves a wide variety of interactions between clinicians and pregnant mothers: exchanging pregnancy and birth information, educating, screening for abnormalities and complications, monitoring mother/baby and providing continuous care, and preparing for childbirth and motherhood.^3^,^4^ PNC additionally provides opportunities for the detection and treatment of diseases, by providing interventions in a timely fashion, promoting overall wellness, and facilitating informed birth choice.^2^–^8^

Over the last 50 years, the mean age at childbearing has increased, leading to adverse health outcomes related to fertility, maternal complications, and infant outcomes.^9^ The average age of first delivery increased from 23–25 years in the 1970s to 27–29 years in 2017, then to 28–30 in 2022.^9^,^10^ Although some of the increase in maternal age is due to decreases in teenage pregnancies, most of the increase is attributed to social changes, such as effective oral contraceptives, the development of assisted reproductive technology, and women prioritizing their education and careers.^9^,^11^ Women aged 35 or older, medically referred to as of “advanced maternal age,”^12^,^13^ (AMA) are at increased risk of many adverse maternal and child health outcomes, including maternal mortality, miscarriage, preterm labor, gestational diabetes, pre-eclampsia, increased probability of requiring a C-section, gestational hypertension, stillbirth, chromosomal abnormalities, low birthweight, need for neonatal intensive care, and low APGAR score.^12^ This makes adequate PNC even more essential in ensuring the health of AMA mothers and their infants.

Lower maternal age also has important ramifications for health outcomes. Pregnancy and birth complications are the number one cause of maternal mortality in teenage women.^14^,^15^ While on a global level teenage pregnancy (pregnancy in those younger than 19) has been decreasing since the 1970s, rates are higher among those from racial- or ethnic-minority groups^16^, and teenage pregnancy is associated with poverty, less education, and single marital status.^17^–^20^ Previous research has also observed increased risk of inadequate PNC and poor infant outcomes in teenaged pregnant women.^14^,^15^,^21^–^23^

When compared to other states, West Virginia (WV) has some of the poorest health and socioeconomic statistics in the U.S., ranking second-highest in terms of poor population health.^24^ Although overall population health in the state is below the U.S. average, the percentage of expectant mothers receiving inadequate PNC in WV (15%) is consistent with the national average across 2016–2020.^1^ WV has a high rate of teenage pregnancy, with 22.5 per 1,000 births being among those aged 15–19 compared to the national rate of 15.4 per 1,000.^25^ These poor health indices indicate this population may provide valuable insight to PNC improvement. Determining what age groups are most at risk of PNC inadequacy can aid in targeting education and interventions directly to these at-risk groups to improve rates of PNC across the state of WV.

In summary, PNC is vital for the health of mothers and their infants.^3^,^4^ AMA and teenage mothers have increased risk to their health and to their infants.^12^ Given its poorer health outcomes and SES statistics relative to the rest of the U.S., WV is a particularly important place to examine the intersection between maternal age and PNC. Thus, this study seeks to determine if an association between maternal age (<19 years, 20–24 years, 25–29 years, 30–34 years, 35–39 years, and 40 years) and inadequate PNC (< 10 visits) exists in WV and, if so, further understand it.

## METHODS

### Subjects

This study used de-identified data from a state-mandated surveillance tool called Project WATCH. This tool collects data on all infants born in WV and their mothers with the goal of identifying infants who are at greatest risk for health and developmental problems (26). Project WATCH, therefore, identifies characteristics of newborns at higher odds of infant mortality and health problems.^27^,^28^ This study used data from May 2018 to March 2022, resulting in a de-identified data sample of 70,724 birthing persons. Gender was not asked of participants; thus, although we may occasionally refer to these individuals with female descriptive characteristics such as “mothers”, we understand that not all of our individuals may identify with those terms. This study was deemed exempt by the West Virginia University Institutional Review Board.

### Measurements

The main outcome variable for this study is inadequate PNC. Project WATCH collects PNC visits as a binary variable and categorizes fewer than 10 PNC visits as inadequate care, and 10 or more PNC visits as adequate care. While there are many ways to define inadequate PNC, this method was chosen due to previous research based on American College of Obstetricians and Gynecologists guidelines stating that 10 or 11 PNC visits is optimal.^29^ The cutoff established by Project WATCH data was previously determined by receiver operating curve (ROC) analysis that 10 PNC visits optimized the sensitivity and specificity for PNC visits and infant mortality, and also determined that the strength of the bivariate associations were stronger with fewer than 10 PNC visits being the cutoff for inadequate PNC.^30^

The main exposure variable of interest for this study was maternal age. In the dataset, maternal age is a continuous variable in years, but it was categorized into five groups based on previous research: teenage (19 or younger), 20–24, 25–29, 30–34, 35–39, and 40 years or older.^31^–^34^ The aged 25–29 category was selected as the referent group, consistent with previous research^31^–^34^ and because the average age of first birth is 28–30 years.^9^,^10^

Sociodemographic variables and other confounding variables were guided by prior research^2^–^8^,^15^,^18^,^19^,^22^,^23^,^35^,^36^ and include maternal race (white, black, Asian, Hispanic, multiracial, and other); maternal education (≤8^th^ grade, 9^th^ grade, 10^th^ grade, 11^th^ grade, 12^th^ grade, and some college or greater); payment method (private insurance, WV Medicaid, self-pay, other, and unknown); smoking status during pregnancy (yes/no); substance use status during pregnancy (yes/no); geographic region (Right from the Start program regions 1–8 and out of state^37^); and parity (0, 1, 2, and 3 or more). Project WATCH collects data on substance use in pregnancy as a binary response (yes/no), which is assessed using self-report, medical records, and/or positive drug test. Affirmative responses were indicated if pregnant individuals used of any of the following: opioids, stimulants, sedatives-hypnotics, phencyclidine (PCP), cannabinoids, gabapentin, or antidepressants.^38^ For geographic region, the most rural region with the fewest birthing hospitals was selected as the referent group. This was Region 4, which contains six counties but only one birthing hospital in the central eastern part of the state (all WV geographic regions are shown in **Fig. 1** in the Additional Files section).^37^

### Data Analysis

Statistical analyses were conducted in SAS version 9.4 (SAS Institute, Cary NC). Basic descriptive statistics were performed on all variables. Frequencies and valid percentages were calculated for categorical demographic characteristics and covariates for first the full sample, and then stratified by adequate/inadequate PNC. Chi-square statistics with accompanying *p*-values were calculated to determine significance of categorical associations to inadequate PNC. Logistic regression analysis was used to examine the bivariate relationship between maternal age and adequacy of PNC, and multiple logistic regression analysis was used to examine the relationships between maternal age and adequacy of PNC with covariates. Adjusted and unadjusted odds ratios (OR) were calculated for having inadequate PNC by maternal age category using maternal age group 25–29 as the reference group. Accompanying 95% confidence intervals (CI) are also presented. Any confounding variables found to be related to PNC at a conservative alpha ≤ 0.20 were adjusted for in the final model.

## RESULTS

This study used data from a population-based cohort of all births in WV from May 2018–March 2022. Study characteristics are presented in Table 1, below, and demonstrate that 92.6% of the population was white; 50% had some college education; 46.9% had private insurance; and 44.2% had WV Medicaid. Among all the births in WV during that period, 11.0% of pregnant persons received inadequate PNC. Of the persons giving birth, 6.3% were aged 19 years or younger, of which 13.9% received inadequate PNC. The data show that 28.9% were aged 20–24 years, of which 11.2% received inadequate PNC. Close to one-third, 32.8%, were aged 25–29, of which 11.3% received inadequate PNC. Just under one-quarter of persons giving birth, 22.5%, were aged 30–34, of which 11.1% received inadequate PNC. Persons aged 35–39 made up 9.6% of this population, of which 12.2% received inadequate PNC. Finally, 1.9% of the population were persons aged 40 years and older, of which 13.7% received inadequate PNC.

For all births in the state of WV, the unadjusted odds of receiving inadequate PNC is significantly higher for the maternal age groups of 19 and younger (aOR:1.3, CI:(1.2,1.4)), *p* < 0.0001), 35–39 (OR:1.1, CI:(1.0,1.2), *p* = 0.02), and 40 and older (OR:1.2, CI:(1.1,1.5) *p* = 0.01) when compared to the reference group of 25–29 years of age (Table 2). When adjusting for significant covariates, the adjusted odds of receiving inadequate PNC slightly attenuated but remained higher for pregnant persons aged 19 years and younger (aOR: 1.3, CI:(1.1,1.4), *p* < 0.0001), 35–39 (aOR: 1.1, CI:(1.0,1.2), *p* = 0.05), 40 and older (aOR: 1.3, CI:(1.1,1.5), *p* = 0.01 ) compared to persons aged 25–29 years.

While not the primary focus of this study, an exploration of significant confounding variables also provides interesting information for WV. Although only representing a small minority of the rural Appalachian state (<1%), those who identified as Hispanic were 1.5 times (CI:(1.2,1.9), *p* = 0.0003) as likely to receive inadequate PNC compared to those who identified as white. Birthing persons receiving an eighth-grade education or less (aOR:3.3, CI:(2.7,4.0), *p* < 0.0001); ninth-grade education (aOR:2.1, CI:(1.8,2.4), *p* < 0.0001); tenth grade (aOR:1.7, CI: (1.6,2.0), p <0.0001); eleventh grade (aOR:1.7, CI:(1.5,1.9), *p* < 0.0001); and twelfth grade (aOR:1.3, CI:(1.2,1.4), *p* < 0.0001) had increased odds of receiving inadequate PNC compared to those who had at least some college education. Increased parity was also a risk factor for increased odds of receiving inadequate PNC for one child (OR:1.2, CI:(1.1,1.3), *p* < 0.0001); two children (OR:1.4, CI:(1.3,1.5), *p* < 0.0001), and three or more (OR:1.7, CI:(1.5,1.8), *p* < 0.0001) when compared to birthing persons with no previous children. Payment method also proves to be a significant risk factor; when compared to individuals with private insurance, birthing persons who self-pay for their care are at almost six times the odds of receiving inadequate PNC (OR:5.9, CI:(4.9,7.1), *p* <0.0001). Birthing persons with WV Medicaid (OR:1.9, CI:(1.8,2.0), *p* <0.0001); birthing persons with a payment method classified as ‘other’ (OR:1.6, CI:(1.5,1.8), *p* < 0.0001); and those whose payment method is unknown (OR:1.9, CI:(1.5,2.3), *p* <0.0001) are also at significantly increased odds when compared to those with private insurance. Birthing persons who use substances were at 3.6 times increased odds of inadequate care (OR:3.6, CI:(3.4,3.8), *p* < 0.0001); and birthing persons who smoke were at almost two times increased odds (OR:1.9, CI:(1.8,2.0), *p* < 0.0001). When compared to Region 4 (i.e., the six most rural central and eastern counties with only a single birthing hospital), birthing persons who live in Region 1, southeast counties (OR:0.7, CI:(0.6,0.8), *p* < 0.0001); Region 2, southwest counties (OR:0.6, CI:(0.6,0.7), *p* < 0.0001); Region 3, the capital urban region and surrounding counties (OR:0.6, CI0.5,0.7), *p* < 0.0001); and Region 5, central northwest counties (OR:0.9, CI:(0.7,1.0), *p* = 0.0127) were all at significantly decreased odds of inadequate PNC. Birthing persons who live in Region 6, the northern panhandle counties (OR:1.2, CI:(1.1,1.4), *p* = 0.0078), and Region 7, the north central counties (OR:1.3, CI:(1.1,1.4), *p* < 0.0001), are at significantly increased odds of receiving inadequate PNC when compared to those who live in Region 4.

## DISCUSSION

This study adds to the limited literature on maternal age and inadequate PNC in a rural Appalachian state, WV. The results show that 12% of pregnant persons in WV receive inadequate PNC per the dataset definition of inadequate care determined by Umer et al.^30^ This number is slightly less than the U.S. average of inadequate prenatal care (~15% in 2020^2^), although differences may also be related to a less conservative measure of inadequate PNC in this study.

The results also demonstrate that teenaged pregnant persons (aged 19 years or younger) and pregnant persons of AMA are at increased odds of receiving inadequate PNC over the course of their pregnancies. Literature states that teenagers and mothers of AMA are at increased odds of poor maternal and infant outcomes^12^–^15^,^20^,^39^ While some literature exists showing teenaged mothers have poorer inadequate prenatal care,^3^,^4^,^8^,^36^,^40^–^42^ this study adds to the literature showing increased odds of inadequate PNC in birthing persons aged 19 or younger, aged 35–39, and aged 40 and older relative to the referent group of 25–29 years. While the literature on PNC inadequacy across maternal age groups is scarce, there is literature showing that pregnant teens^43^ and women with increased parity^44^ are more likely to receive inadequate PNC. Increased parity could possibly explain why it was found AMA pregnant persons were at increased odds of inadequate care.

Next, this analysis demonstrates interesting trends for covariates in terms of inadequate PNC. Consistent with the literature,^41^ lower educational attainment–in this case, high school education and less—was associated with inadequate PNC relative to having at least some college education. Also consistent with previous literature,^44^ increased parity is associated with increased inadequacy of PNC. Pregnant people identifying as Hispanic (<1% of the study’s population) have increased odds of inadequate PNC, which is in line with other publications suggesting an area of policy improvement for the state of WV and on a national scale.^45^–^47^

The relationship between insurance status and inadequate PNC was consistent with previous literature, as well.^7^,^8^,^41^,^42^,^48^–^51^ Compared to those with private insurance, individuals with Medicaid have increased odds of inadequate PNC, as well as those who self-pay, have other insurance types, and whose insurance type is unknown. The analysis also shows results consistent with previous literature^2^ that individuals who smoke and who use substances during pregnancy have increased odds of inadequate PNC compared to those who do not.

Last, high rates of inadequate PNC were observed in the northern regions after accounting for covariates. While several counties in these regions do not have birthing hospitals and are fairly rural (e.g., Barbour, Doddridge, Harrison, Preston, Taylor, Tucker Tyler, Wetzel), this was a surprise as the selected comparison region (Region 4, including Braxton, Fayette, Greenbrier, Nicholas, Pocahontas, and Webster counties) had only a single birthing hospital for six of the most rural counties in the state. When looking at unadjusted rates, Region 4 had the highest inadequate PNC in the state (14.6%); however, the other northern regions of 6 and 7 also had high inadequate PNC (14.0%).

### Limitations

Although the study appropriately models population-level data to demonstrate a relationship between PNC and maternal age, there are some limitations to consider. First is the lack of information regarding other potential confounding variables, such as household income, marital status, support within the household, and access to affordable childcare. Not being able to control for these factors may introduce information bias into the study. Second, the results may not be generalizable outside of the state. However, though these data are specific to WV, the study does demonstrate the value of further research on a wider scale to determine association between maternal age and PNC in other states or even nationally. Finally, the definition of PNC was previously defined in the Project WATCH dataset and cannot be adjusted to fit other indices of PNC; this limits inferences to other definitions of inadequate PNC. Despite its limitations, this study bolsters understanding of the relationship between multiple maternal age categories and inadequate PNC in the state of WV.

## IMPLICATIONS

This study has many implications for public health, particularly within the state of WV. Determining what age category group (19 or younger, aged 20–24 years, 25–29 years, 30–34 years, 35–39 years, and 40+ years) of pregnant persons are at increased odds for receiving inadequate PNC in WV can aid in directing more targeted research or prevention measures into improving education and access to PNC for pregnant persons of that age group. This research can spur future research on barriers to inadequate PNC utilization faced by these at-risk demographic groups. This research can also aid in improved education on the importance of PNC to teen mothers in the state. This information contributes to the broader literature, as well—specifically to literature on maternal age and its effect on PNC adequacy.

**SUMMARY BOX**

**What is already known about this topic?**

PNC is vital for the health of mothers and their infants.^3^,^4^ AMA and teenaged mothers have increased odds of poor health outcomes, as do their infants.^12^ WV is a particularly important place to examine this intersection between maternal age and PNC because of the poorer health and SES statistics across this rural Appalachian state.

**What is added by this report?**

This report adds vital information on what age groups are at increased odds of receiving inadequate PNC.

**What are the implications for future research?**

The implications of this report’s findings can aid in more targeted research and prevention measures to certain at-risk groups. This research could also help improve PNC education overall in the state of WV and across the Appalachian Regions

## Figures and Tables

**Table 1:** Study Characteristics of Persons Who Gave Birth to All Infants Born in the State of WV, May 2018 March 2022 (n = 70,724)

Variables	Total Frequency (%)	Inadequate PNC Frequency (%)	Adequate PNC Frequency (%)	Chi-square *p*-value
**Maternal Age**				< 0.0001
19 or younger	4,347 (6.3%)	603 (13.9%)	3,744 (86.1%)	
20–24	19,969 (28.9%)	2,244 (11.2%)	17,725 (88.8%)	
25–29	22,677 (32.8%)	2,554 (11.3%)	20,123 (88.7%)	
30–34	15,534 (22.5%)	1,728 (11.1%)	13,806 (88.9%)	
35–39	6,638 (9.6%)	815 (12.3%)	5,823 (87.7%)	
40+	1,349 (1.9%)	183 (13.6%)	1,166 (86.4%)	
**Race**				< 0.0001
White	64,516 (92.6%)	7,403 (11.5%)	57,113 (88.5%)	
Black	1,798 (2.6%)	235 (13.1%)	1,563 (86.9%)	
Asian	416 (0.6%)	33 (7.9%)	383 (92.1%)	
Hispanic	679 (1.0%)	117 (17.2%)	562 (82.8%)	
Multiracial	783 (1.1%)	128 (16.4%)	655 (83.7%)	
Other	1,476 (2.1%)	228 (15.5%)	1,248 (84.6%)	
**Maternal Education**				< 0.0001
8th Grade or Less	556 (0.8%)	206 (37.0%)	350 (63.0%)	
9th Grade	1,088 (1.5%)	300 (27.6%)	788 (72.4%)	
10th Grade	2,095 (3.0%)	514 (24.5%)	1,581 (75.5%)	
11th Grade	3,607 (5.1%)	795 (22.0%)	2,812 (78.0%)	
12th Grade	27,839 (39.5%)	3,795 (13.6%)	24,044 (86.4%)	
Some College	35,244 (50.0%)	2,478 (7.0%)	32,766 (93.0%)	
**Parity**				< 0.0001
0	21,325 (30.2%)	1,772 (8.3%)	19,553 (91.7%)	
1	20,291 (28.7%)	2,022 (10.0%)	18,269 (90.0%)	
2	13,345 (18.9%)	1,641 (12.3%)	11,704 (87.7%)	
3 or more	15,713 (22.2%)	2,852 (18.2%)	12,861 (81.9%)	
**Payment Method**				< 0.0001
WV Medicaid	31,206 (44.2%)	5,409 (17.3%)	25,797 (82.7%)	
Private	33,167 (46.9%)	1,843 (5.6%)	31,324 (94.4%)	
Self Pay	680 (1.0%)	239 (35.2%)	441 (64.9.%)	
Other	4,537 (6.4%)	666 (14.7%)	3,871 (85.3%)	
Unknown	1,075 (1.5%)	126 (11.7%)	949 (88.3%)	
**Smoking Status**				< 0.0001
Yes	15,252 (21.6%)	3,992 (26.2%)	11,260 (73.8%)	
No	55,394 (78.4%)	4,264 (7.7%)	51,130 (92.3%)	
**Substance Use**				< 0.0001
Yes	9,574 (13.5%)	3,341 (34.9%)	6,233 (65.1%)	
No	61,149 (86.5%)	4,992 (8.2%)	56,157 (91.8%)	
**Region**				< 0.0001
1	1,628 (8.9%)	694 (11.0%)	5,620 (89.0%)	
2	1,952 (10.7%)	721 (8.9%)	7,402 (91.1%)	
3	2,415 (13.2%)	782 (8.7%)	8,212 (91.3%)	
4	1,093 (6.0%)	631 (14.8%)	3,644 (85.2%)	
5	1,324 (7.2%)	602 (11.6%)	4,568 (88.4%)	
6	1,173 (6.4%)	663 (14.0%)	4,058 (86.0%)	
7	3,563 (19.5%)	1,938 (14.0%)	11,921 (86.0%)	
8	1,608 (8.8%)	889 (14.6%)	5,195 (85.4%)	
Out of State	3,562 (19.5%)	1,251 (10.6%)	10,599 (89.4%)	

**Table 2:** Unadjusted and Adjusted Odds Ratios of Maternal Age Category by Prenatal Care (PNC) Inadequacy in the State of WV, May 2018 March 2022 (n = 70,724)

Model	Predictor	Odds Ratio (95% CI)	Chi-Square	*p*-value
**Unadjusted Model**	**Maternal Age Category**			
	19 or younger	1.3 (1.2,1.4)	24.0	<0.0001
	20–24 years	1.0 (0.9,1.1)	0.1	0.9348
	25–29 years	1		
	30–34 years	1.0 (0.9,1.1)	0.2	0.6733
	35–39 years	1.1 (1.0,1.2)	5.2	0.0226
	40 and older	1.2 (1.1,1.5)	6.7	0.0098
**Adjusted Model**	**Maternal Age Category**			
	19 and younger	1.3 (1.1,1.4)	17.7	<0.0001
	20–24 years	1.0 (0.9,1.1)	0	0.9977
	25–29 years	1		
	30–34 years	1.1 (1.0,1,1)	1.5	0.2178
	35–39 years	1.1 (1.0,1.2)	3.7	0.0545
	40 and older	1.3 (1.1,1.5)	6.8	0.0093
**Covariates**	**Race**			
	White	1		
	Black	1.0 (0.9,1.2)	0.1	0.7792
	Asian	1.3 (0.9,1.8)	1.6	0.1992
	Hispanic	1.5 (1.2,1.9)	13.1	0.0003
	Multiracial	1.2 (1.0,1.5)	3.6	0.0578
	Other	1.1 (1.0,1.4)	2.5	0.112
	**Maternal Education**			
	8th Grade or Less	3.3 (2.7,4.0)	137,0	<0.0001
	9th Grade	2.1 (1.8,2.4)	84.2	<0.0001
	10th Grade	1.8 (1.6,2.0)	78.1	<0.0001
	11th Grade	1.7 (1.5,1.9)	103.0	<0.0001
	12th Grade	1.3 (1.2,1.4)	69.5	<0.0001
	Some College	1		
	**Payment Method**			
	Private Insurance	1		
	WV Medicaid	1.9 (1.8,2.0)	24.0	<0.0001
	Self-Pay	5.9 (4.9,7.1)	346.0	<0.0001
	Other	1.6 (1.5,1.8)	66.0	<0.0001
	Unknown	1.9 (1.5,2.3)	33.2	<0.0001
	**Smoking Status**			
	No	1		
	Yes	1.9 (1.8,2.0)	440.1	<0.0001
	**Substance Use**			
	No	1		
	Yes	3.6 (3.4,3.8)	1669.0	<0.0001
	**Region**			
	Region 1	0.7 (0.6,0.8)	38.4	<0.0001
	Region 2	0.6 (0.5,0.7)	55.2	<0.0001
	Region 3	0.6 (0.5,0.7)	68.0	<0.0001
	Region 4	1		
	Region 5	0.9 (0.7,1.0)	6.2	0.0127
	Region 6	1.2 (1.1,1.4)	7.1	0.0078
	Region 7	1.3 (1.1,1.4)	17.5	<0.0001
	Region 8	1.0 (0.9,1.2)	0.1	0.7792
	Out of State	0.9 (0.8,1.0)	1.8	0.1766
	**Parity**			
	0	1		
	1	1.2 (1.1,1.3)	26.8	<0.0001
	2	1.4 (1.3,1.5)	54.6	<0.0001
	3 or more	1.7 (1.5,1.8)	155.3	<0.0001
